# Review of Plasmonic Nanocomposite Metamaterial Absorber

**DOI:** 10.3390/ma7021221

**Published:** 2014-02-14

**Authors:** Mehdi Keshavarz Hedayati, Franz Faupel, Mady Elbahri

**Affiliations:** 1 Nanochemistry and Nanoengineering, Faculty of Engineering, Institute for Materials Science, Christian-Albrechts-Universität zu Kiel, Kaiserstrasse 2, Kiel 24143, Germany; E-Mail: mke@tf.uni-kiel.de; 2 Chair for Multicomponent Materials, Faculty of Engineering, Institute for Materials Science, Christian-Albrechts-Universität zu Kiel, Kaiserstrasse 2, Kiel 24143, Germany; E-Mail: ff@tf.uni-kiel.de; 3 Institute of Polymer Research, Helmholtz-Zentrum Geesthacht, Max-Planck-Str. 1, Geesthacht 21502, Germany

**Keywords:** perfect absorber, metamaterials, plasmonic, nanocomposite

## Abstract

Plasmonic metamaterials are artificial materials typically composed of noble metals in which the features of photonics and electronics are linked by coupling photons to conduction electrons of metal (known as surface _lasmon). These rationally designed structures have spurred interest noticeably since they demonstrate some fascinating properties which are unattainable with naturally occurring materials. Complete absorption of light is one of the recent exotic properties of plasmonic metamaterials which has broadened its application area considerably. This is realized by designing a medium whose impedance matches that of free space while being opaque. If such a medium is filled with some lossy medium, the resulting structure can absorb light totally in a sharp or broad frequency range. Although several types of metamaterials perfect absorber have been demonstrated so far, in the current paper we overview (and focus on) perfect absorbers based on nanocomposites where the total thickness is a few tens of nanometer and the absorption band is broad, tunable and insensitive to the angle of incidence. The nanocomposites consist of metal nanoparticles embedded in a dielectric matrix with a high filling factor close to the percolation threshold. The filling factor can be tailored by the vapor phase co-deposition of the metallic and dielectric components. In addition, novel wet chemical approaches are discussed which are bio-inspired or involve synthesis within levitating Leidenfrost drops, for instance. Moreover, theoretical considerations, optical properties, and potential application of perfect absorbers will be presented.

## 1. Introduction

The present reviews start with a short introduction to the plasmonic absorption while spanning the development of perfect absorbing structures. The general theoretical consideration for metamaterials absorber will be presented in Section 2. Section 3 focuses on the design, fabrication and characterization of plasmonic nanocomposite and its implementation as a building block for the design of perfect black absorber (base on gold, copper and silver). Moreover, the similarity and performance of the different metamaterial absorber also will be presented in Section 3. Discussion of selective innovative approaches for highly absorber devices is the focus of Section 4. The last Section 5 presents the prospects and future potential of metamaterial absorbers.

### 1.1 Plasmons and Plasmonics

One of the prime applications of nanostructures dates back to more than three millenniums ago [1], when the ancients used metallic particles in ceramic matrix (most probably not aware of the dimension of particles) for decoration purposes. The trace of this old-fashioned nanotechnology nowadays can be seen in many museums and historical sites worldwide in the form of colorful tiles [2], lusters [3], glasses [4], *etc.* The real origin of the coloration was unknown up to the early 20^th^ century, when theoretical studies shed light into this uncovered optical phenomena [5] which is known today as plasmonics [6] (photon-electron coupling). Surface plasmons are waves that spread over the surface of a conductor. Hence, changing the surface structure alters the light-plasmon interaction, the phenomenon which provides the potential for emerging new photonic devices [7].

In general, materials with a negative real and small positive imaginary dielectric constant are capable of sustaining a surface _lasmon resonance (SPR). For materials whose dimensions are far below the sub-wavelength, in particular nanoparticles, _lasmon oscillates locally around the particle and hence it is called localized surface _lasmon resonance [8]. In other words, the conduction electrons in the nanoparticles (NPs) move all in phase upon excitation with incidence light and polarize the particle surface [9]. Since the electrons are displaced from their equilibrium state, the redistribution tendency of surface charge applies a restoring force on the disordered electrons and results in oscillation with a certain frequency, known as _lasmon resonance frequency [10]. Therefore, a field builds up inside the particle while establishing a dipolar field on the outer surface of particle. This strongly enhanced near field around the NPs which considerably increase their absorption and scattering cross section is the primary reason immense attention to plasmonic nanoparticles [11]. In fact, the optics has been revolutionized within the last few decades owing to the plasmonic nanostructures and consequently, design of sub-diffraction opto-electronic devices is nowadays possible.

### 1.2. Energy and Materials

The oil crisis in the late 70s [11] redrew attention toward plasmonic materials, although not for luxury purposes but rather as a new alternative absorber for solar collectors where light trapping is highly desired [12]. As discussed in the last section, the near field enhancement of plasmonics could be also applicable in solar cells to reduce the thickness (accordingly the material cost) of photovoltaic devices significantly [13]. Ceramic-metals alloy (also known as cermet [12]) were in use since then as a method of choice for solar thermal collector. These categories of composites withstand the harsh environment and do not degrade at high temperature. To enhance their absorption, graded coating (out of composite) on a metallic substrate is applied where the refractive index decreases monotonically in passing from the substrate to the surface [12,14]. In other words, traditional Rayleigh configuration for low reflection has been used in order to achieve low reflection loss and high absorption, simultaneously. Even thought cermet coating works well for realization of high absorption in visible range, the film is bulky and thick and hence does not match with the miniscule modern devices.

However, fast growth of nanotechnology in the last two decades and the development of metamaterial in the beginning of the 21^st^ century [15] unlocked a new door to design of miniaturized and efficient absorber. In analogy with cermet coating, nanofabricated structures replaced the traditional top composite layer (in a triple layer absorber) in order to achieve a similar goal but on a smaller scale. In this novel approach, the performance was similar in the sense of absorption intensity but the band-width was narrow [16–18]. This originated from the fact that the new developed coating material (conventional metamaterial) was designed for a single frequency, and to reach multiple wavelengths, multiple types of structures in a single system are required. The latter is rather costly, time consuming and relatively hard, especially when it is intended to absorb short wavelength light (visible or UV). Hence, development of alternatives or redesign of traditional cermet seems necessary as a way to maintain high efficiency while down-scaling the size to a few 10ths of nanometers.

### 1.3. Highly Absorbing Structures: Metal Nanostructure and Films

Beside the variety of applications routed from individual nanoparticles (NPs) [19,20], interacting NPs provide greater localization of the electromagnetic field [21,22] which broaden the usefulness of nanoscale metallic particles. Due to the localization of surface _lasmon, the ensemble of NPs, which are no more distant from each other than the diameter of each individual particle, could strongly trap the incident light in a sub-wavelength scale (gap between the NPs) which could create a huge localized field. The spectrum of such groups of NPs is determined by the interaction between the individual localized surface _lasmon (LSP) resonances. The magnitude of the confined field and its frequency depend significantly on the shape, size and the space between NPs [23].

Similar to particles’ ensemble, but in a simpler situation, strong interaction of electric field can happen when a metallic particle (or collection of particles) is situated atop of a metal surface. This system can be named as coupling of localized and delocalized _lasmon resonance [24]. In other words, when a dipole is located in proximity of a conducting layer (mirror), in addition to the dipole-dipole interaction [25–28], dipole interaction with the induced image in the mirror influences the resonance [28–30] (optical response).

**Figure 1.**
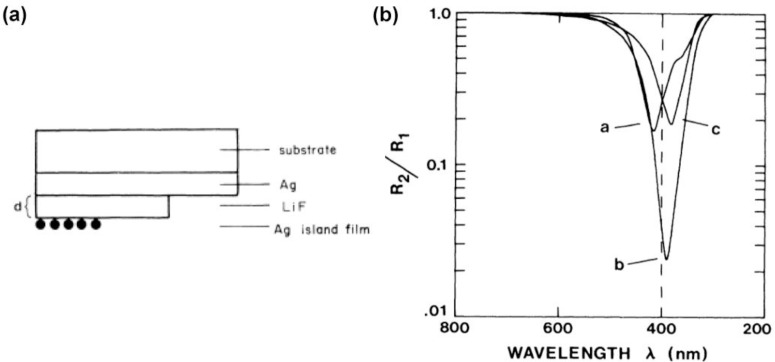
(**a**) Cross section of the three-layer sample geometry used in [31]; (**b**) Measured specular reflectance of silver-island samples R2 (normalized by Ag-LiF reflectance R1) as a function of wavelength for three different values of spacer-layer thickness *d*. The minima near 400 nm correspond to absorption by the silver islands. Curve *a*, *d* = 8 nm; curve *b*, *d* = 27 nm; curve *c*, *d* = 36 nm. (Reprinted with permission from [31]. Copyright 1982 American Physical Society).

Study of the dipole-conductive film dated back to late 80s when Holland and Hall [31] analyzed the interaction of silver or gold particles (dipoles) and silver film (conducting surface) which were separated by LiF layer (Figure 1a). They considered the observation of frequency shift as a universal feature of the dipole-surface interaction caused by the coupling between the dipole and its own image in the metal surface as it is shown in Figure 1b [31]. Following this work, Borensztein *et al.*, improved the calculation method of earlier work by taking into account the interactions of a silver sphere with both its own image field reflected from the conducting surface and with the image field of all the other spheres [32]. Cesario *et al.* [33] studied a very similar system except that the top layer was lithographically fabricated gold particles and the spacer was a 10 nm Indium Tin Oxide layer. They observed appearance of two separate peaks. They attributed one of the peaks to the localized surface _lasmon of the nanostructures and their own interaction. While the second peak referred to the surface _lasmon polariton trapped at the gold–glass interface which was excited by the energy transfer of LSP of the structure to the surface _lasmon polariton (SPP) of the film (grating coupling) [33]. There have been so many similar works in the mentioned combination where the frequency shift and coupling of _lasmon resonance were studied [29,34–37]. However, one of the first reports of highly absorbing film-particle absorber was theoretically shown by Papanikolaou in which silver spheres (90 nm in diameter) are sited on top of silver film (40 nm thick) [38]. Although the aim of the work was not realization of a highly absorbing system, but rather to study the effect of effect of particle arrangement on optical properties, the author achieved this outcome.

**Figure 2.**
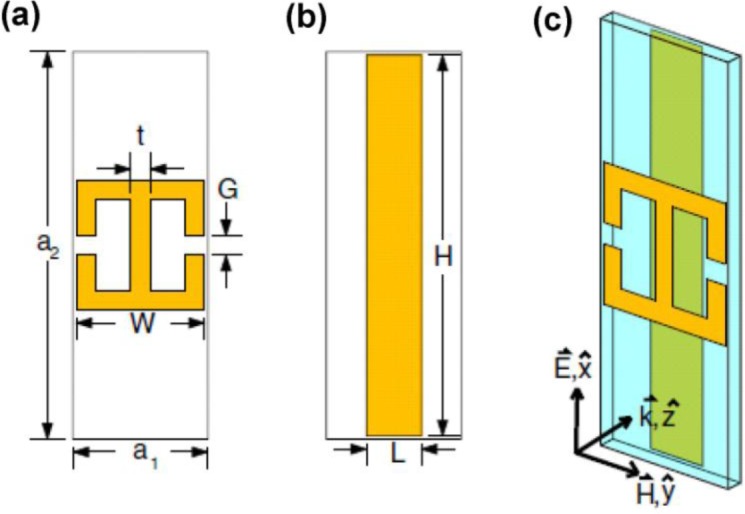
Electric resonator (**a**) and cut wire (**b**). Dimension notations are listed in (**a**) and (**b**). The unit cell is shown in (**c**) with axes indicating the propagation direction [16]. (Reprinted with permission from [16]. Copyright 2008 American Physical Society).

Although the currently recognized field of metamaterials absorber looks novel, it has basically the old geometry of metallic particle-film (dipole-conductive metal) which has been extensively studied in the last few decades. In other words, patterned micro (nano-) structures replace the older particles and molecules due to the advances in nanofabrication techniques. Nevertheless, considering the high absorption in metamaterials as a new objective (despite early consideration of absorption as a limiting factor), broadened the application of this field. The work of Padilla and colleagues is the start of metamaterials perfect absorber [16] or more precisely, renaissance of electric dipole (resonator)-film interacting structures. In that early work, the authors showed that metamaterials consisting of two standard split ring resonators connected by an inductive ring parallel to the split-wire, placed in a distance from a cut-wire, could absorb light completely in certain wavelength ranges. The geometry of their designed metamaterial absorber is illustrated in Figure 2 [16].

This work inspired the researchers working with metamaterials to reexamine the optical structures that had been developed up until that time. Accordingly, a tremendous number of perfect absorber structures were designed and developed (theoretically and experimentally) which realize almost unity absorption at different frequencies. However, the principle behind all the methods was the same and the structures were mainly composed of three layers; top metallic structure and substrate with a dielectric interlayer. Moreover, fabrication of the top structured layers was carried out mainly by lithography. The latter fact makes large area coverage difficult and limits the down-scaling (of nanostructure) below 50 nm because lithographically production of smaller features is rather complicated (For details of the development of metamaterials electromagnetic absorber, see the recent review by Padilla and co-workers [39]).

## 2. Theoretical Consideration

The theory of the metamaterial absorber is presented thoroughly in the progress report by Padilla and co-workers [39] and herein we briefly summarize the general theoretical background in this section.

Based on Fresnel equation, the reflectivity at the air interface of a medium with refractive index *n* ( $n=\sqrt{\mu .\varepsilon }$ [40]) for transverse electric (TE) and transverse magnetic I polarized waves are as follow [39]:

$$\begin{array}{l} R_{TE}={\left|\frac{\text{cos}\theta -\frac{\sqrt{n^{2}-{\text{sin}}^{2}\theta }}{\mu_{r}}}{\text{cos}\theta +\frac{\sqrt{n^{2}-{\text{sin}}^{2}\theta }}{\mu_{r}}}\right|}^{2}\\ R_{TM}={\left|\frac{\varepsilon_{r}\text{cos}\theta -\sqrt{n^{2}-{\text{sin}}^{2\;}\theta }}{\varepsilon_{r}\text{cos}\theta +\sqrt{n^{2}-{\text{sin}}^{2}\theta }}\right|}^{2} \end{array}$$

in which θ is the angle of incidence, *n* is the refractive index, ε and μ are permittivity and permeability of the medium (metamaterial), respectively. In case of normal incidence angle (θ *=* 0), the above equation reduces to [39]:

$$\begin{array}{l} R_{TE}={\left|\frac{1-\frac{n}{\mu_{r}}}{1+\frac{n}{\mu_{r}}}\right|}^{2}\\ R_{TM}={\left|\frac{\varepsilon_{r}-n}{\varepsilon_{r}+n}\right|}^{2} \end{array}$$

If it is intended to have no reflectivity for both polarisations, both of the above terms should equal to zero which is equivalent to μ_*r*_ = ε_*r*_. This means impedance of the metamaterial should match that of air in order to reduce the reflection loss down to zero. According to Kirchhoff’s rule, the sum of the transmittance *T*, reflectance *R*, and absorbance *A* should be equal to 1 in the absence of scattering and diffraction [41]. Taking both mentioned facts into consideration, it can be concluded that if the impedance of a metamaterial matches to that of the free space and the medium is opaque (zero light transmission) the light can be absorbed totally [42]. Note that if the medium is not sufficiently thick and not enough lossy, the wave which is bounced back off the base mirror can reflect back into free space [39]. Although the satisfaction of the impedance matching is rather hard, by using an optically thick metal plate, the zero transmission condition can be provided. Therefore, the challenge is to design and manufacture a structure with impedance matching the free space for a single or wide range of frequency. Usually, in multi-layer structures, impedance matching is accomplished either by using anti-reflection coating or by a dielectric film (certain thickness) flanked by partially reflecting mirrors (*i.e.*, Fabry-Perot interferometer) [43]. In both mentioned cases, an additional lossy medium is required in order to absorb the light and dissipate the incidence energy.

It is known that metals are lossier in high frequency, in particular at optical realm, due to electron transitions from the filled *d* bands into the *SP* conduction bands (absorption) [44]. However, at lower frequency (longer wavelengths), one can assume that most metals act as a perfect conductor with small loss, since the corresponding ohmic loss fraction (the ratio of the skin depth over wavelength) is only 0.1% or less [43]. This means that for the metamaterials designed for infrared (IR), the source of the main loss is the dielectric. This is the main reason why lossy dielectric is incorporated in metamaterial design. In other words, lowering the reflectivity by impedance matching is not sufficient and presence of lossy materials is obligatory for realization of high absorptivity. In contrast to low frequency, contribution of metallic absorption (e.g., ohmic loss and surface _lasmon decay) is more than dielectric when the operating frequency is Near-infrared (NIR) or visible. Therefore, absorption within the metallic part in metamaterial absorbers for visible frequency reduces the role of dielectric absorption, and hence relatively thinner dielectric is sufficient for high frequency purposes.

By taking into consideration all the mentioned facts, recently a new perfect absorber was designed and fabricated which is orders of magnitude thinner than conventional cermet while its absorption, band-width and intensity is surpassing that of conventional metamaterial absorber and traditional cermet. Here, the top layer is replaced by ultra-thin (~20 nm) plasmonic nanocomposite made of metal nanoparticles dispersed randomly in polymeric (or generally dielectric) matrix. In spite of the early consideration of graded refractive index layers as beneficial method for higher absorption, it is shown that thin film of a highly dispersive material (plasmonic nanocomposite) [45] with a high refractive index contrast to the second layer (interlayer) could give rise to perfect absorption of light in a broad range of frequency from deep UV [46] up to visible and NIR [47,48]. The key feature of this metamaterial absorber is reversing the traditional arrangement of layers where the refractive indices ascend in consecutive layers. Impedance matching of the medium to free space, multi-reflection of light between the layers and light trapping and absorption by the tiny metallic particles enable realization of almost unity absorption of light in wide range of spectrum from UV to NIR.

## 3. Metal-Dielectric Nanocomposites with Tailored Plasmonic Response

Many approaches have been reported to prepare metal-dielectric nanocomposites containing metallic nanoparticles embedded in a dielectric organic or ceramic matrix due to their unique functional properties with hosts of applications (for recent reviews see [49,50]). For the present application of metal-dielectric composites in plasmonic metamaterial absorbers, two revere restrictions apply. First, a high filling factor of the metallic nanoparticles close to the percolation threshold is required to take advantage of the interaction of _lasmon resonances localized at individual nanoparticles. Second, large area coverage is indispensable in most applications which rules out electron beam lithography and rather calls for self-organized formation of the nanostructures. This is why vapor phase deposition techniques are particularly attractive for tailoring the nanostructure and the resulting properties [49]. Vapor phase deposition, *inter alia*, allows excellent control of the metallic filling factor and its depth profile as well as the incorporation of alloy nanoparticles with well-defined composition [51]. The metallic nanoparticles typically form via a self-organization during co-deposition of the metallic and matrix components due to the high cohesive energy of the metals and the low metal-matrix interaction energy [49,52]. Various methods such as sputtering [53,54], evaporation [55], and plasma polymerization [56] have been applied for the deposition of the matrix component, while the metallic component has mostly been sputter-deposited or evaporated. Moreover, gas aggregation cluster sources were utilized to obtain independent control of filling factor and size of the embedded nanoparticles [57]. Examples of plasmonic metal-dielectric composites are given in [30,46,47,49,51,56,58,59].

### 3.1. Fabrication Procedure

For all types of nanocomposites discussed in this review, the same fabrication procedure was used [47] which is summarized as follows. Magnetron sputtering was carried out for deposition of all the films and composite of various types and compositions. The machine was a cylindrical custom-build stainless steel vacuum chamber located in a cleanroom. Argon was used as inert gas for sputtering. During the deposition process, samples were rotating with a constant speed by means of a speed controlled motor in order to assure the uniformity of the deposited film.

For metallic film preparation (e.g., gold or silver), a direct current (DC) magnetron was used where the distance between the center of sample holder and head of the magnetron was 14 cm. The initial base pressure of the chamber and sputtering pressure were 10^−6^ mbar and 2.5 × 10^−3^ mbar, respectively. For sputtering of the insulating materials (Polytetrafluoroethylene (PTFE), TiO_2_ and SiO_2_), radio frequency (RF) sputtering was applied to neutralize the charge accumulation at the surface of the substrate during the deposition [60].

For nanocomposite deposition, co-sputtering method was applied. Both targets (DC and RF sources) were installed opposite of each other except for the case of co-sputtering of copper and PTFE where the targets were not opposing. For preparation of nanocomposite with different filling factor, the rate of each of the materials (metal and dielectric) were determined separately at the same pressure and gas flow rate which was used for co-deposition. The filling factor was estimated by considering the equivalent film thickness of each component within the time scale of deposition. Then, the ratio of metallic film to overall thickness was considered as the filling factor of the nanocomposite. To confirm the mentioned estimation, Energy-dispersive X-ray spectroscopy (EDX) analysis of the typical composite with different filling factor was performed which agrees quite well with the value determined by thickness consideration.

### 3.2. Results and Discussion

Since the type of metallic constituent in the nanocomposite is the most influential parameter on the optical properties of the system, in the following, three different nanocomposite perfect absorbers based on gold, copper and silver will be presented.

#### 3.2.1. Gold Nanocomposite

Though gold is one of the known primeval noble metals and has been the subject of investigation in science for ages [61], the current state-of-the-art nanofabrication methods reorient the studies of this metal for a host of new applications in electronics and optics. Au NPs are among the most stable metal nanoparticles with some unique features and properties such as size-related electronic, optical and magnetic properties as well as application in catalysis and biologic systems [61]. In the field of plasmonic materials, the gold (in particular in nanoparticles form) is also the leading building block not only because of its stability but also due to its _lasmon resonance and unique optical properties in visible. Forasmuch as the mean free path in gold and silver is 50 nm, particles smaller than this size do not experience any bulk scattering and surface effect is dominating. Therefore, the light in resonance with the surface _lasmon oscillation causes the free-electrons in the metal (*d* electrons in silver and gold) to oscillate. Since the resonance occurs at the surface, it is called surface _lasmon resonance (SPR). Consequently, any effect which changes the surface geometry of the particle (e.g., size or shape) causing a shift in the electric field density on the surface which results in the alteration of oscillation frequency of the electrons (*i.e.*, SPR shift) [62]. Changing the surrounding environment of the NPs could also affect the resonance frequency which is the basic principles of plasmonic sensor (for details see the review by Stewart *et al.* [63] and the references therein).

**Figure 3.**
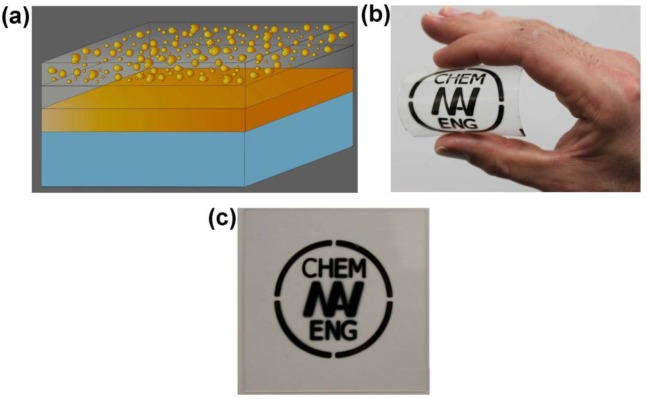
(**a**) Schematic of the perfect absorber structure fabricated by sputtering. The thickness of the layers from top to down are (*i.e.*, nanocomposites, SiO_2_ spacer, and the gold mirror) 20, 25, and 100 nm, respectively. Perfect absorber (blackbody) coated via a mask on (**b**) flexible polymer foil and (**c**) glass. This example shows the potential of the coating for application on different substrates. (Figure 3b reprinted with permission from [47]. Copyright 2011 Wiley-VCH Verlag GmbH & Co. KgaA).

It is well known that the interacting metallic particles and film could considerably absorb light. Inspired with earlier works on metal-polymer nanocomposite [58,64] and recent developments in the field of metamaterials, the authors of the present article found that ultra-thin nanocomposite atop of dielectric coated metal film could result in complete absorption of light in broad spectrum. Similar to the older works, a three-layered structure was developed. However, the main difference was the use of ultra-thin metal-dielectric nanocomposite (highly dispersive material [45]) as a top roof film (Figure 3a). Gold was selected as a prime metallic constituent of the proposed structure due to its great stability and unique optical properties. Experimental data verified that 20 nm nanocomposite (Au-SiO_2_) deposited on 25 nm SiO_2_ coated gold film (100 nm) is the optimum condition for realization of broad-band perfect absorber in visible frequency. It was found that the volume fraction (filling factor) of the gold in composite significantly alters the optical response of the designed metamaterials. By optimization, it was found that 40% filling factor is the optimum value for high absorption in a wide range of wavelengths making the surface appearance black (Figure 3b,c). The authors postulated that the huge absorption originated from several factors as follows. Impedance matching in these metamaterials as well as dipole-image (polarized particle and its image in the base mirror) interaction, which causes an electromagnetic confinement in the spacer layer, alleviates reflection [47]. The broad resonance of the absorber stem, due to the fact that the broad Mie resonance of the nanoparticles ensembles (which originates from the large size distribution of the particles with random shapes), and the _lasmon polariton of the base metal film overlap. A study of the optical data measured at a higher angle of incidence confirmed the mentioned reasoning in which the broad plasmonic resonance peak splits into two peaks along with a slight drop in absorption in grazing incidence [47].

Changing the thickness of the spacer layer (interlayer) revealed that the dipole-image interaction is one of the reasons for light trapping in the nanocomposite perfect absorber. For instance, thickening the interlayer results in a drop of the absorption intensity, which can be interpreted as weaker dipole-image interactions due to weaker coupling [47].

**Figure 4.**
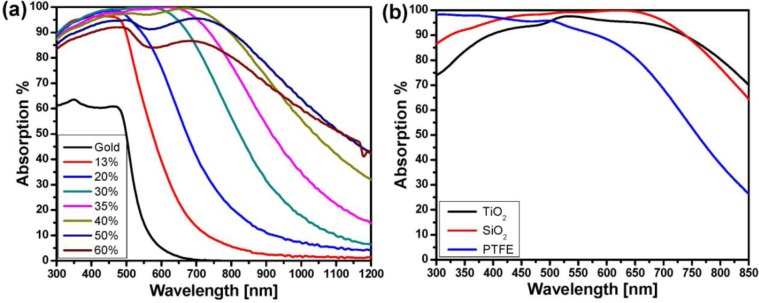
(**a**) Absorption spectra of a 20-nm Au-SiO_2_ nanocomposite with different filling factor sputter-deposited on a 100-nm gold film which has been coated with a 25 nm SiO_2_ spacer layer. The absorption calculated by measuring the reflection (at a 6° angle of incidence) using *A*% = 100% − *R*% while assuming the scattering is negligible. (**a**) Absorption spectra of a 20-nm Au-SiO_2_ (circles), 20-nm Au/-TiO_2_ (triangles) and 20-nm Au-PTFE (squares) nanocomposite on a 100-nm gold film with a 25 nm SiO_2_ at a 6° angle of incidence (Figure 4b reprinted with permission from[47]. Copyright 2011 Wiley-VCH Verlag GmbH & Co. KgaA).

Tuning the optical response of any metamaterials is desired. It was shown that alteration of the filling factor could provide the possibility of adjusting the resonance position. As it is shown in Figure 4a, the absorption peak either shifts towards the NIR when increasing or towards the blue when decreasing the filling factor [47].

Since the resonance of the plasmonic structure is strongly influenced by any changes in the surrounding environment, the peak position of absorption in the plasmonic nanocomposite perfect absorber is also altered by changing the matrix. Figure 4b depicts the absorption of the absorber with three different matrices of the composite (PTFE, TiO_2_, and SiO_2_). In the case of the matrix with lower refractive index (PTFE) compared to the SiO_2_, the resonance blue shift, while having a higher refractive index material (TiO_2_), shifts the peak to a longer wavelength. It seems that the retardation effect of the higher refractive-index matrix (TiO_2_) on the resonance of the metallic particles is shifting the absorption band to a longer wavelength while the polymer matrix with less dielectric constant (than that of silicon dioxide matrix) provides the condition for higher resonance frequency.

#### 3.2.2. Copper Nanocomposite

Copper has been in use by humans from early civilization since people could simply cold-hammer native copper for building tools. Similar to gold, copper soon found its way toward decorative applications (beside its extensive types of mechanical usefulness). The ruby color of some glasses nowadays is attributed to the existence of nanoparticles of copper immersed in a silica matrix [65,66]. In the field of metamaterials, and in particular, perfect absorbers, copper is a promising candidate due to its significant loss in visible range, though it has been implemented and used for realization of high absorber devices in other frequencies, too.

One of the first copper metamaterial perfect absorbers was demonstrated by Soukoulis and co-workers [67] in both numerical simulations and experimental measurements for GHz in the form of chiral metamaterial. A material is considered to be chiral if it lacks any planes of mirror symmetry. They were inspired by early works on chiral metamaterials [68] where significant loss is originated from dielectric loss in the FR-4 board at microwave [67]. Hence, they selected lossy elements (copper) in their design to achieve higher absorption. After Soukoulis’s work, numerous perfect absorbers which have copper as their constituent were presented for GHz. The majority of the works were based on the three-layer absorber where a dielectric is sandwiched between two metallic structures and/or films. In the majority of the available literatures, FR4 (lossy dielectric) film were used for copper absorber [69–76]. Indeed, the role of dielectric seems to be more significant than the metal itself. Based on the two studies in which the dielectric contribution to the absorption is analyzed, the role of dielectric loss in absorption is considerably larger than that of the ohmic losses in metal [76,77].

Although the majority of the works were limited to the three-layer system with lossy dielectric, there have been some more innovative designs and proposed structures in which other possible influential parameters are changed in order to improve the absorption efficiency in terms of band-width and _lasmon_ty. One of the unique works in the field of copper base metamaterial absorber is the work by Sun *et al.* [78] which showed that by means of destructive interference, broadband absorption of light can be realized. They showed that the choice of proper refractive index dispersion enables the designer to produce a consecutive anti-reflection which may widen the bandwidth of the absorber significantly. To achieve such a goal, multilayer of split-ring resonators (SRRs) with different dimension stacked over each other to provide a dispersive refractive index required for anti-reflection (high absorption). In other words, their proposed structure shows high absorption not because of resonant loss of SRRs but rather due to the anti-reflectivity of the dispersive coating [78]. Integrating resistors into resonators while maintaining an impedance-matched material at normal incidence was another innovative method used by Gu *et al.* [79] in order to have a broader absorption band. With that strategy, they could realize an absorber with a peak absorption of 99.9% at 2.4 GHz, and a full width at half maximum (FWHM) of 700 MHz. Similarly, to broaden the bandwidth of the metamaterial absorber, Cheng *et al.* [80] incorporated lumped elements (resistance and capacitance) into a typical three-layer structure (dielectric substrate sandwiched with metal split-coin resonators (SCR)) by welding. In such a system, which is in analogy with an RLC circuit, the incidental EM energy can be converted into electric energy in the circuit, and then electric energy can be subsequently consumed by lumped resistances [80].

The trends to turn metamaterial devices from passive performers to active (tunable) ones increased considerably. Accordingly, the same interest holds for absorbers based on copper elements. Wen *et al.* [81] incorporated VO_2_ to the traditional multilayer absorber to enable the resonance _lasmon_ty by temperature variation. Since the vanadium dioxide phase transition from metallic to insulating occurs around 330 K, its refractive index changes correspondingly. Therefore, the resonance condition inside the structure would change by heating or cooling the sample at this temperature range, giving rise to the realization of a tunable perfect absorber [81].

In spite of all the reported highly absorbent metamaterials in which the top layers are patterned metallic structures, Shu *et al.* [82] recently showed numerically that the triple absorber consisted of metallic film (non-structured) as the top and bottom layers surrounding a dielectric film, which could act as the perfect absorber for visible and IR frequency. However, their design is based on Fabry-Perot interferometer principles and therefore the thickness of the interlayer and its refractive index is relatively high. The ease of fabrication of such a class of absorber is beneficial, but its narrow bandwidth and its rather bulky thickness would be its limiting factors for application in nano-optics.

**Figure 5.**
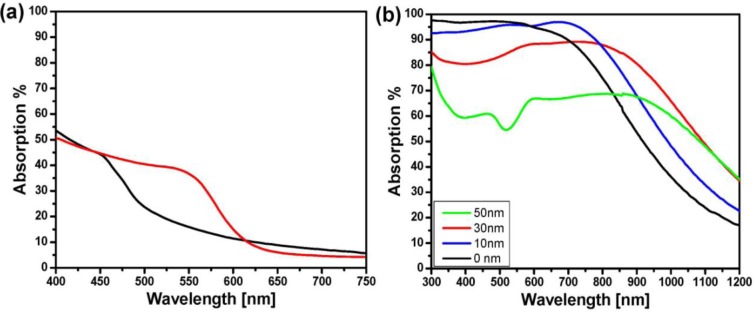
(**a**) Absorption spectra of 20 nm Cu-PTFE composite (black) with a sputtering ratio of 2.0 on 20 nm PTFE on glass in comparison of 100 nm copper film (red). (Figure 5b reprinted with permission from [48]. Copyright 2011 Springer Science and Business Media); (**b**) Absorption spectra of a 20-nm Cu-PTFE nanocomposite sputter-deposited on a 100-nm copper film which has been coated with different thickness of spacer layer. The absorption calculated by measuring the reflection (at a 6° angle of incidence) using *A*% = 100% − *R*% while it assumes that scattering is negligible.

Generally, the metamaterial absorbers made of copper suffer from one or some of the following limitations; fabrication complexity, cost of production, narrow band of resonance and angular/polarization sensitivity. In our recent work, we tried to fabricate an ultra-thin triple-layer perfect absorber with copper as its metallic constituents in which a straightforward fabrication process is used and a wide absorption band with marginal sensitivity to the angle of incidence demonstrated for visible and NIR [48]. As discussed earlier, metal-dielectric nanocomposite in a stack with metallic and dielectric film could confine the light and results in broad-band perfect absorber. Analogous to gold nanocomposite, copper nanocomposite were fabricated but by using an organic matrix. In the copper-based absorber, the bandwidth is broader and given that the copper is cheaper than gold, the new developed absorber could be more cost effective for practical application. The average absorption intensity of copper absorber in visible frequency is above 97%, which makes it, to the best of our knowledge, the most intense reported broadband plasmonic perfect absorber so far. Note that the absorption of bare copper film or single layer Cu-PTFE nanocomposite are rather poor and their average intensity in visible is below 30% [48] as shown in Figure 5a.

As dielectric, PTFE was used which has a low refractive index (~1.3) to assess the role of dielectric type on the optical properties of absorber. In spite of Au-SiO_2_ absorber where 25 nm SiO_2_ interlayer showed the best performance, 20 nm spacer layer of PTFE resulted into the maximum absorption in copper system [48]. Thickening the spacer layer to 50 nm whilst the other parameters kept the same, the absorption intensity drops considerably (Figure 5b). Additionally, in spite of gold absorber which was shown in the previous section, deposition of nanocomposite on bare copper (with no spacer layer) could lead also to very high absorption intensity (Figure 5b). These evidences prove that both coupling and interference contribute to the high absorption of such a structure. However, it seems that copper particles act as stronger light absorber than gold which can be the cause of the high absorption of structure even without any spacer film.

**Figure 6.**
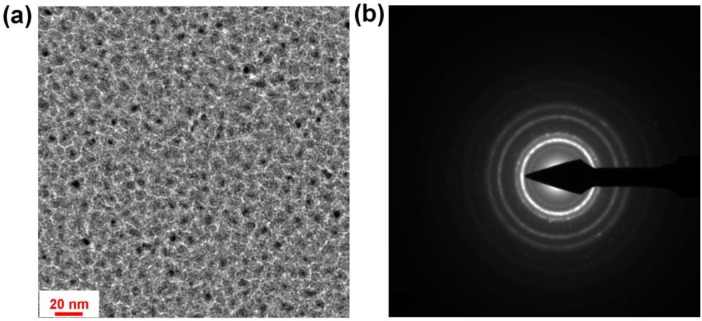
(**a**) Top-view TEM image of the near percolated copper-PTFE nanocomposite; (**b**) The selected area diffraction pattern of the structure shown in (**a**).

The absorption intensity and broadness of copper perfect absorber is greater than that of its gold counterpart. The transmission electron microscopy (TEM) image of typical Cu-PTFE composite used in [48] is shown in Figure 6a. The electron diffraction pattern (Figure 6b) shows that Cu similar to Au is formed as nanocrystallites in the matrix, as confirmed by the rings with diffuse intensity representing the various Miller planes [83]. It seems that the differences in the absorption properties of copper and gold base absorber is routed mainly from the fact that copper is more lossy in visible frequency than gold. Nevertheless, the overall behavior of the two mentioned systems shows some similar tendencies where the absorption intensity in entire visible spectrum is high.

In addition to the dipole-image interaction and light trapping between the particles gap, one can explain macroscopically the optical behavior of such metamaterials by interference theory [42]. In other words, not only _lasmon coupling but rather interference and multi-excitation of resonance because of the mirror nature of the base layer contribute to the high absorption (low reflection) of the multi-stacks. By changing the thickness of the spacer layer, the resonance frequency and intensity of the peak vary which shows that not only the _lasmon absorption of the particles but also the interaction of the particles and the film contribute to the absorption of the current metamaterial.

Even though huge absorption was realized in copper based metamaterials’ absorber, the structure and optical properties vary by time mainly due to the probable oxidation of particles via interpenetration of oxygen to the particles surface. Generally, the main difficulty in utilizing copper nanoparticles is their inherent tendency to oxidize in ambient conditions. Applying different barrier layer is known as the major solution to that issue [84]. However, the polymeric (PTFE) matrix which was used as the dielectric could not provide the efficient protective layer and avoid copper NPs oxidation. Further works are needed to encapsulate copper nanoparticles in an oxygen impenetrable matrix to guarantee the long term stability of the final device.

#### 3.2.3. Silver Nanocomposite

Silver in different forms being used for curing of burns, wounds and several bacterial infections for thousands of the years. In the current century and thanks to the advances in nanofabrication techniques, the range of silver applications has broadened, especially silver NPs which are now known as a potential antimicrobial agent [85]. In spite of extensive work on silver nanostructure as the main metallic constituents of metamaterials [86–92], metamaterial perfect absorbers are mainly composed of gold. It seems that gold have been in use in the mentioned field because of ease of fabrication, stability and high absorption in the middle of visible spectrum. In spite of high damping losses of gold and copper, silver is known as the lowest damping metal in the visible frequency [93] and hence consideration of silver as the constituent of perfect absorber sounds unreasonable. In other words, silver has been implemented in metamaterials for high frequency because of its low loss [94–98].

It is known that the interband transition from occupied *d* states to unoccupied *p* and *s* states above Fermi level appear at 310 nm and 350 nm in bulk silver, respectively. However, for silver nanostructures, such electron transitions could occur above 350 nm wavelengths and depend on nanostructure geometry [99]. Therefore, high optical absorption in silver particles is likely to occur at 350 nm wavelengths and above. On the other hand, silver’s reflection is close to unity over the whole visible region and hence the absorption of optically thick Ag film is usually below 5% in that region. In comparison with bare silver film, nanocomposite shows higher absorption and its intensity is around 30% in the visible frequency. In spite of the expected low absorption of silver in visible frequency, our experimental data demonstrated that the high absorption span for the UV up until the green part of spectrum can be realized by silver base plasmonic metamaterial absorber [46]. This is coming from the fact that the resonance of plasmonic materials moves to longer wavelengths when the dielectric constant of the adjacent environment enlarges. This is more prominent if the particle is in proximity to a metallic substrate. Particularly, as a dielectric encloses the metallic nanoparticle, the induced screening charges on the metal–dielectric boundary reduce the _lasmon excitation energy resulting in a red-shifting of the resonance. Likewise, for nanoparticles ensembles, the dielectric materials screen and weaken the interaction between the NPs and lessen the shift of the coupled _lasmon [100]. Accordingly, the absorption band of the silver absorber moves to the visible range due to the interaction of the composite and the base silver mirror. It is worth mentioning that part of the red-shift originated from the particle dipole which is anti-symmetrically coupled to its image inside the silver mirror (substrate) [24].

In analogy with copper perfect absorber system, low reflectivity of the silver absorber is partially routed from the interference [101]. In such layered stacks, the Fabry-Perot cavity is built-up between the top composite and the bottom mirror, and results in a strong interference of the incident and the reflected wave. Specifically, the bounced-back rays from the mirror destructively interfere with the direct reflection from the top (air-composite) interface. If the thickness of the spacer layer is properly selected, the reflected waves cancel each other out which results in negligible reflection. Given that the base silver film is thicker than the skin depth, no light transmits through the layers and thus perfect absorption is achieved [46].

One of the major differences of the silver absorber and the two other types of absorbers (gold and copper based absorbers) which were discussed above is the appearance of an absorption dip at higher frequency (around 325 nm). This dip cannot simply stem from transmission of silver film at 320 nm (5% transmission) because the intensity drops around 30% which is much more than the probable absorption loss due to transmission. The dip is routed from overlapping of two other resonances. One is the surface _lasmon resonance of silver film excited either by diffraction via particles or from the roughness of the film itself. The other is the plasma frequency of silver which appears at 320 nm. In other words, at high frequency, two mentioned absorption peaks appear while their center resonance is apart. Therefore, the gap between the two peaks appears as a dip of absorption [46].

**Figure 7.**
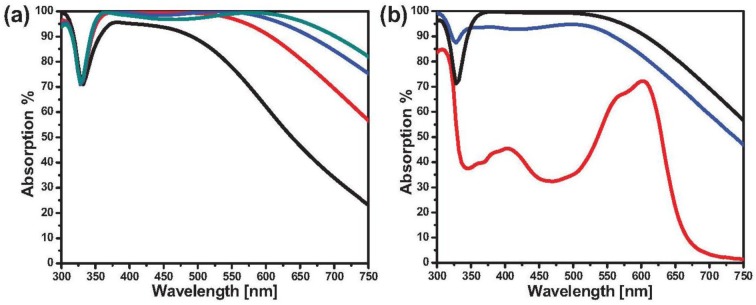
(**a**) Absorption spectra of silver-SiO_2_ nanocomposite with 15 nm (black curve), 20 nm (red curve), 25 nm (blue curve), and 30 nm (green curve) thickness deposited on 15 nm SiO_2_ coated silver mirror; (**b**) Absorption spectra 200 nm silver film coated with 40 nm polystyrene-SPO composite (red curve) and 20 nm silver-SiO_2_ nanocomposite with 42% filling factor deposited on 10 nm (blue curve) and 15 nm (black curve) SiO_2_ film. The organic film is UV illuminated prior to measurement. (Reprinted with permission from [46]. Copyright 2014 American Institute of Physics).

The effect of filling factor and thickness of the film or composite on the optical properties of silver perfect absorber was in accordance with the copper or gold system. The only difference came from the initial peak position which was discussed above. For instance, by increasing the thickness of the composite (while keeping the other parameters constant), the absorption band can be widened extensively. Indeed, metamaterial silver absorber which spans the whole visible frequency can be made by increasing the thickness of nanocomposite with 46% filling factor to 30 nm (Figure 7a).

The interestingly high absorption of the silver system in deep UV range shows its potential as UV protection film. Comparative study of the absorption performance of silver nanocomposite absorber with typical UV absorber (Spirooxazine molecules) showed that the absorption intensity of the presented metamaterials is higher than the inorganic absorber (Figure 7b). Hence, one can see the potential application of perfect absorber as a highly efficient UV protective layer [46].

**Figure 8.**
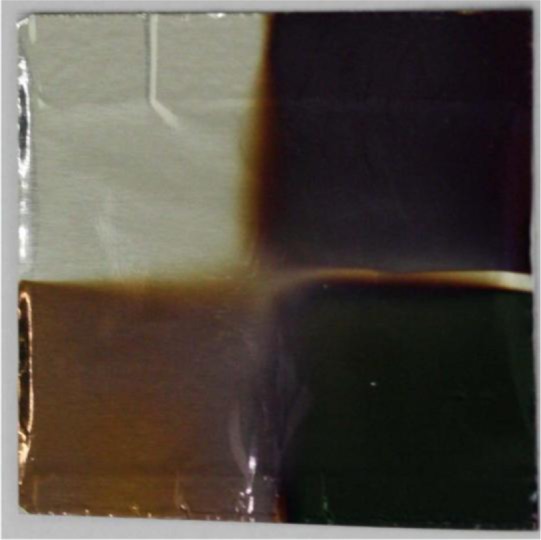
True photograph of 50 nm gold nanocomposite with (13%), (20%) and (30%) filling factor on aluminum foil resulting in different color. The white are (top left) is the bare aluminum foil.

In short, nanocomposite perfect absorber (*i.e.*, nanocomposite-dielectric-metal film stack) shows almost unity absorption in a broad range of frequencies with marginal angular dependency. The absorption peak position and intensity can be tuned by changing the type of nanocomposite, filling factor and the host matrix, and the thickness of the layers which demonstrate the flexibility of the proposed method from both a fabrication and application point of view. The implementation of perfect absorbing structures on a variety of substrate is a new idea which could be implemented into the new generation of thin film solar cells for clothing (textile industry) [102]. Accordingly, we showed that nanocomposite with high filling factor can be deposited even on aluminum kitchen foil, turning it into a black absorber or any other color (Figure 8). This idea can push the field of metamaterials absorber one step forward for novel energy applications.

## 4. Innovative Design of Light Weight Broadband Nanocomposite Perfect Absorbers

As discussed above, majority of metamaterials absorbers (narrow or broadband) consist of three or multiple layers out of metals and dielectric assembled in a way to provide light confinement either by electric/magnetic resonance or via interference and localization. Moreover, physical vapor deposition (PVD) is the prime technique for production of metamaterials absorber for high frequency. Nonetheless, innovative design for realization of broadband perfect absorber have been developed while it is not made through PVD nor designed by triple layers.

**Figure 9.**
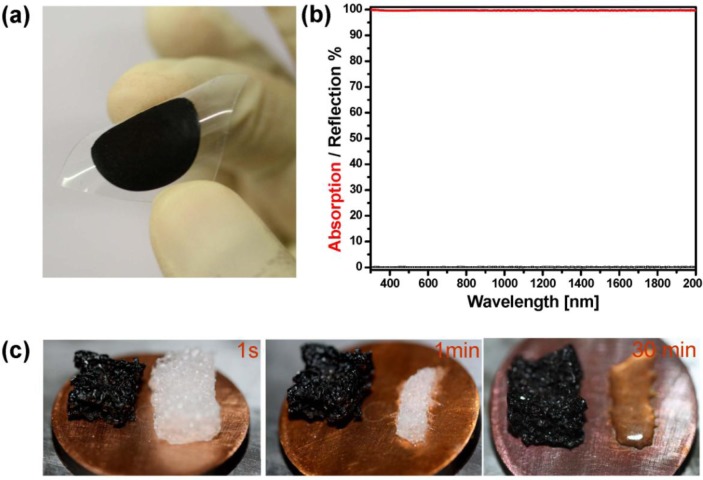
(**a**) A flexible polymeric substrate coated with the black spongy gold; (**b**) Reflection and absorption spectra of black spongy gold in the visible and near-infrared frequency; (**c**) Heating stages of coated (black) and neat foam (white) on a hot plate after **(bottom-left)** 1 s, **(bottom-middle)** 1 min and **(bottom-right)** 30 min at 150 °C. (Reprinted with permission from [104]. Copyright 2013, Nature Publishing Group).

It is known that ensemble of nanoparticles can strongly confine the light. This trapping is more intensified when the particles’ inner-distance shrinks down to the diameter of the particles. Porous metals are among those structures which provide the small inter particles distance and enable localization of electromagnetic field (via localized surface _lasmon resonance). Hence, the tuning of the resonance is directly correlated with the size at dimension of the porosity and the structure itself [103]. This fact was the base of the new design of perfect absorber which has been recently developed by Elbahri and co-workers [104]. They use the Leidenfrost drop to create a nanoporous gold hybrid structure as well as black plasmonic foam, which absorb the whole visible and NIR electromagnetic waves resulting in a very broadband, perfect plasmonic absorber (Figure 9).

When a water drop touches a plate which is hotter than the boiling point of the drop, the part in contact with the substrate vaporizes and the drop levitates on its own vapor. Interestingly, remnant solid is left on the surface over which the drop has levitated. Based on Elbahri’s group finding, overheating, thermal gradients and charge separation are fundamental to Leidenfrost condition. In addition to the great possibility of nanofabrication under the Leidenfrost condition such as nanoparticles formation, coating *etc.*, the authors demonstrated that such an approach can be used for fabrication of 3D metamaterial broadband absorber (400–2500 nm) in a very simple, cost effective and an environmentally friendly manner (Figure 9a,b). In this approach, a drop of (1 mL HauCl_4_, 20 mM + 700 mL Sod. Citrate 1% + 150 mL NaOH 0.5 M, pH~8.5) was placed on a preheated hot plate (with a constant temperature of 270 °C) which provide a suspension of the desire black porous structure in less than a minute. The black suspension can turn a flexible polymeric substrate to a super absorber by a simple casting method (Figure 9a). To realize a macro-scale three dimensional porous metamaterial, a commercial packaging polymer foam is introduced in a levitated black pool out of the porous gold. Thanks to the dynamic covering potential of Leidenfrost drop, the foam was coated with the metallic spongy structures and a millimeter size black 3D metamaterials is realized (Figure 9c). In such a complex polymer-metal structure, the suppression of light reflection is attributed to consolidation of light scattering by the sample roughness, localized and de-localized excitation of plasmons within and on the surface of pores [105], as well as light trapping inside the gaps. Although broadband absorbers are critical in energy harvesting applications, for more effective use of solar energy it is desirable to develop cost-effective, durable and lightweight systems [106] with improved ability to absorb solar radiation energy particularly at wavelengths below 3 μm (c.f. Figure 9b) [107]. This chemically developed metamaterial absorber can withstand high temperatures, which demonstrates its potential application in energy collecting purposes. Figure 9c shows the black coated foam on a hot plate in comparison to uncoated foam. It can be clearly seen that the black metamaterial could stand high temperatures in which the neat foam melts.

**Figure 10.**
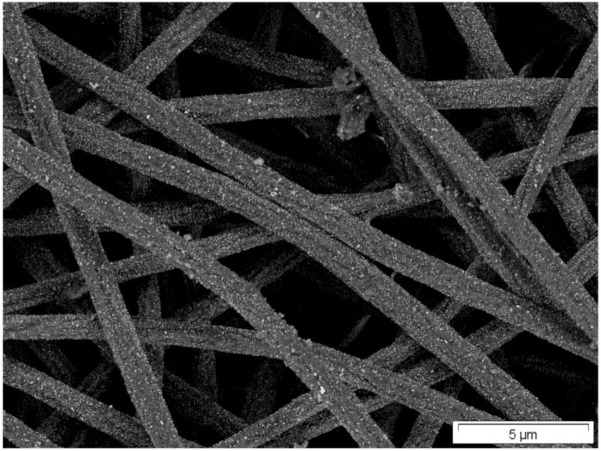
Formation of a bionanohybrid structure through adsorption of Au nanoparticles onto the BSA/PANGMA nanofibers. (Reprinted with permission from [110]. Copyright 2012 Wiley-VCH Verlag GmbH & Co. KgaA).

**Figure 11.**
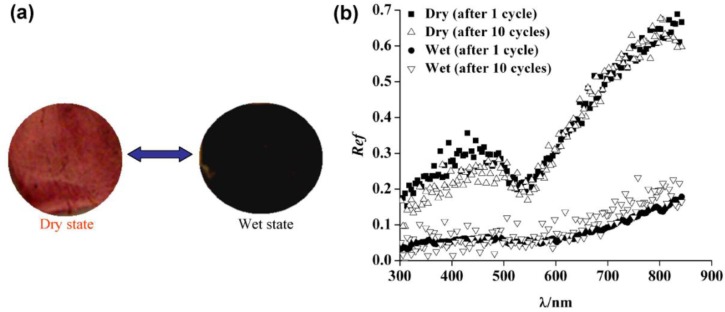
(**a**) Tunable coloration of the surface of the membrane at dry (red) and wet (black) states; (**b**) The reflection spectra of the nanocomposite in dry and wet (completely water soaked) states. (Reprinted with permission from [110] Copyright 2012 Wiley-VCH Verlag GmbH & Co. KgaA).

The biologically inspired fabrication approach is another novel and cost effective method for development of 3D metamaterials perfect absorber. Biological materials are inherently and naturally multifunctional and even smart. For instance, chameleons are well-known for their ability to change color. This stems from the fact that chromatophores (organocell) could either spread the pigment particles all over the cell or concentrate them into a small lump [108]. A quite similar concept is applied for humidity based color change (light blue to black) in *Cryptoglossa verrucosa*. The color phases are formed by “waxfilaments” that spread from the tips of miniature tubercles that cover the cuticle surface [109]. Elbahri and co-workers [104] mimicked the beetles to achieve a broadband and lightweight, perfect absorber using nanocomposites as a standalone matrix. Recently, they have shown a macroporous membrane consisting of polymeric nanofibers and proteins able to filter out tiny nano-scaled particles present in aqueous solutions. A nanofluid (*i.e.*, a colloidal suspension of metal nanoparticles in water) can pass through a macro-porous, nano-fibrous membrane unless the membrane’s nanofibers are bio-functionalized by a globular protein. It was found out that the biofunctional agent (bovine serum albumin) could undergo a conformational change thereby capturing all the metal nanoparticles during the filtration process. Accordingly, a novel method for bio-nanocomposite fabrication has been introduced (Figure 10) wherein the surface color changes (Figure 11a) from red to black upon wetting thereby enabling realization of an omni-directional wideband perfect absorber [110].

In dry state, the sample looks red while the average reflection of the sample is about 35%. On the other hand, by wetting the sample, it turns black while showing low reflectivity (Figure 11b) and it acts as a swollen, open, porous, nanostructure foam. The resulting porous structure gives rise to the localization of the incident electromagnetic field (in analogy to the nanoporous structures). Therefore, the sample turns black and absorbs the visible energy [110]. Nevertheless, this bio-nanocomposite structure is in its infancy and further studies are required to explore the mechanism behind the high absorption.

These mentioned examples show that chemically routed fabrication methods could also provide the possibility for design and fabrication of new metamaterial absorbers. However, wet chemical fabrication techniques are less considered and their pros and cons need to be explored.

## 5. Prospects and Future

The field of metamaterial perfect absorbers is still immature. Much more effort must be made to bridge the gap between the lab-scale fabrication and industrial application. Nevertheless, current achievements both in theory and experiments showed the immense potential of this new type of metamaterials for a variety of applications.

As the prime utility of highly absorptive structures, photovoltaic and solar cells are the fields of interest. Very recently, the effect of typical, perfect absorbing structures (*i.e.*, metallic nanostructure and film separated by a dielectric) on the absorbing efficiency of organic photovoltaic materials was investigated. The authors demonstrated numerically that absorption augmentation up to 2.88 can be achieved in a 40-nm-thick P3HT:PC_60_BM film sandwich by Aluminum nanostructure and film due to critical coupling into the magnetic resonant mode [111,112]. Other uses of metamaterial perfect absorbers are in fields where huge light confinement is desired. Li *et al.* [113], making use of such potential of plasmonic absorber, demonstrate its applicability in surface enhanced molecular spectroscopy (SEMS). Due to the localized field within the nanostructure and its _lasmon_ty, the resonance could match the molecular vibrational modes of interest in the analyte which provides the possibility to identify chemical stretches. They showed that by using a cross-shaped nanoscale structure separated from a gold film by alumina, Parylene C molecular vibrational stretches in very thin film can be revealed [113]. Microbolometer thermal sensors is another general application of metamaterial absorbers [114] Aluminum nano-pattern and aluminum plate with SiO_2_ spacer layer was a design demonstrated by Kearney *et al.* [114].

Gigantic field enhancement achieved by perfect absorber can be applied for Raman spectroscopy of single molecules, too. Wang *et al.* [115] showed this in a structure composed of silver particles (average radius size of around 20 nm) dispersed on a surface of SiO_2_ coated silver mirror. In such a structure, in which they called “metasurface”, surface-enhanced Raman scattering (SERS) enhancement factors which is one order of magnitude higher than those of silver nanoparticle islands on glass can be achieved. They attributed this enhancement to the improvement in the coupling between the incident light and _lasmon resonance of the developed metasurface [115].

Cloaking an object by perfect absorber (in reflection mode) is another potential application of this class of material. Alaee *et al.* [116] numerically showed that any object which can be wrapped by a perfect absorber would be cloaked and turn invisible in reflection due to the suppression of back-scattered light from the wrapped object. Their proposed structure is also composed of metallic pattern and film but on a curved surface.

Perfect absorbers designed for low frequency have been shown to be acoustic metamaterials which absorb the airborne sound in the frequency range of 100–1000 Hz. The designed and fabricated structure comprises an elastic membrane decorated with asymmetric rigid platelets. This intelligently designed metamaterial can have a broad range of applications such as reducing the cabin noise in airliners and ships, regulating the acoustic quality of music halls, and environmental noise abatement along highways and railways, amongst others [117].

Similar to other plasmonic structures, application of metamaterial perfect absorbers as a sensor [118] is also promising, in particular when the absorber is narrowband. In all reported works, tri-layer absorber is designed and it is shown that the resonance band of such structures can be tuned upon exposure to different liquids [119,120] or vapor [121] because of the refractive index change of the surrounding environment.

Taking into consideration all the illustrated applicability of metamaterial perfect absorbers, one could see the progress in this new and fast growing field. However, none of the proposed structures have been used in the currently industrialized solar cells or collectors and therefore their long term performance and stability needs to be examined. Moreover, up-scaling of the nano-lithographically fabricated system (which is the major fabrication method in metamaterial) in a cost-effective and reproducible way is also in doubt and requires the invention and development of some new robust and cheaper alternatives. Nevertheless, the future of this field is very bright and mass production and implementation of metamaterial perfect absorbers for everyday life is not out of reach in the current decade.
